# A patient-centered composite endpoint weighting technique for orthopaedic trauma research

**DOI:** 10.1186/s12874-019-0885-7

**Published:** 2019-12-26

**Authors:** Ugochukwu N. Udogwu, Andrea Howe, Katherine Frey, Marckenley Isaac, Daniel Connelly, Dimitrius Marinos, Mitchell Baker, Renan C. Castillo, Gerard P. Slobogean, Robert V. O’Toole, Nathan N. O’Hara

**Affiliations:** 1 Department of Orthopaedics, University of Maryland School of Medicine, 110 S. Paca St., Suite 300, Baltimore, MD 21201 USA; 2 Department of Health Policy and Management, Johns Hopkins Bloomberg School of Public Health, 624 N Broadway, Baltimore, MD 21205 USA

**Keywords:** Best-worst scaling choice experiment, Orthopaedic trauma, Fracture, Study design, Composite endpoint, Weighting technique, Patient-centered care

## Abstract

**Background:**

This study aimed to address the current limitations of the use of composite endpoints in orthopaedic trauma research by quantifying the relative importance of clinical outcomes common to orthopaedic trauma patients and use those values to develop a patient-centered composite endpoint weighting technique.

**Methods:**

A Best-Worst Scaling choice experiment was administered to 396 adult surgically-treated fracture patients. Respondents were presented with ten choice sets, each consisting of three out of ten plausible clinical outcomes. Hierarchical Bayesian modeling was used to determine the utilities associated with the outcomes.

**Results:**

Death was the outcome of greatest importance (mean utility = − 8.91), followed by above knee amputation (− 7.66), below knee amputation (− 6.97), severe pain (− 5.90), deep surgical site infection (SSI) (− 5.69), bone healing complications (− 5.20), and moderate pain (− 4.59). Mild pain (− 3.30) and superficial SSI (− 3.29), on the other hand, were the outcomes of least importance to respondents.

**Conclusion:**

This study revealed that patients’ relative importance towards clinical outcomes followed a logical gradient, with distinct and quantifiable preferences for each possible component outcome. These findings were incorporated into a novel composite endpoint weighting technique.

## Background

A commonly used definition of a composite endpoint in clinical research is the occurrence of any one of several study events of interest [1]. Incorporating multiple endpoints into a single metric increases the number of observed events, can avoid issues pertaining to multiplicity, and thus, may increase statistical power [1–3]. Composite endpoints also enable the inclusion of rare, but clinically important, outcomes; therefore, providing a broader interpretation of the net clinical benefit of a treatment [1].

Composite endpoints have several limitations [4–7]. The treatment effect of an outcome of high importance but low frequency, such as death, may be muted by the inclusion of more common outcomes of lesser importance, such as a superficial infection [4]. Additionally, in studies that analyze composite endpoints using a traditional time to first event analysis or other analyses of frequency that only consider the first event, each study participant can have only one event; therefore, censoring subsequent events biases treatment effects to earlier outcomes. Efforts to address these limitations have included weighting techniques such as those utilizing the Delphi method [8], disability-adjusted life years [9, 10], or hierarchical and global ranking systems [11–14]. However, weighting methods with incorporated patient values specific to the target patient population are lacking [11, 12].

Composite endpoints are becoming increasingly common in orthopaedic trauma research. The objective of this study was to address the limitations related to the use of composite endpoints in orthopaedic trauma research. The primary aim was to quantify the utility and heterogeneity of utility of clinical outcomes common to orthopaedic trauma patients using a Best-Worst Scaling experiment. The secondary aims were to use the patient values derived from the Best-Worst Scaling experiment to develop a patient-centered composite endpoint weighting technique that accounts for multiple events per patient. Finally, we provide one hypothetical clinical trial example and several options for how the weights may be applied in practice.

## Methods

### Study design

A Best-Worst Scaling experiment was used to determine the relative importance of common clinical outcomes to orthopaedic trauma patients. Best-Worst Scaling experiments are a type of choice experiment that were first devised for marketing research but have been more recently applied to healthcare research [13, 14]. Choice experiments assume that any product or service, such as a healthcare treatment or clinical outcome, can be described by its characteristics, or attributes [15]. In a Best-Worst Scaling experiment, respondents are presented with a set of three or more attribute levels and then asked to select the best and worst attribute level in each choice set. The utility of each attribute level is then determined based on the probability of respondents choosing one attribute level over others [16]. The mean utility of each attribute level is then reported relative to a single, common reference level. In this study, the calculated utilities were used to produce a weighting technique accounting for the patient-reported importance of orthopaedic clinical outcomes.

### Attribute development and survey design

**Fig. 1:**
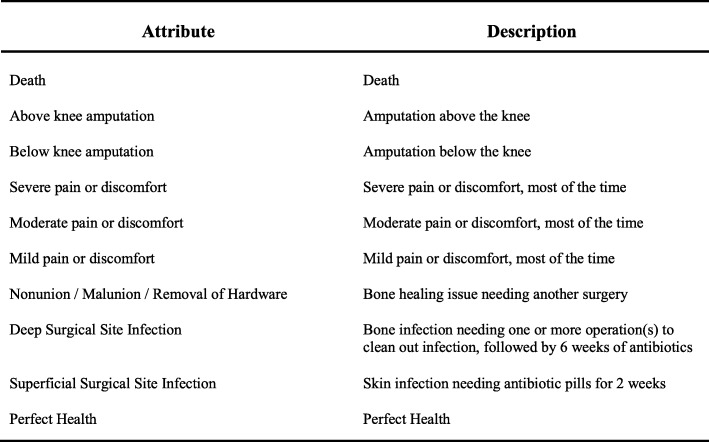
Description of the attribute levels used in the Best-Worst Scaling questionnaire

The study was performed at a single Level-1 trauma center in Baltimore and followed the International Society for Pharmacoeconomics and Outcomes Research conjoint analysis practice guidelines [17]. The attributes used in this study were selected through a combination of quantitative and qualitative methods. A literature review identified common components of composite endpoints used in orthopaedic trauma research [18–21]. Expert consensus was elicited from orthopaedic trauma surgeons at the study location. Finally, semi-structured interviews were conducted with three orthopaedic trauma patients for additional perspective on plausible clinical outcomes. Information gathered from this work informed the final selection of the included attributes and levels deemed most important by our patient and clinician stakeholders. Orthopaedic trauma patient-partners then participated in the development of patient-oriented descriptions of each attribute level. Fig. 1 lists the attributes included in the final Best-Worst Scaling experiment questionnaire. The MaxDiff Design platform in JMP Pro Version 13 (Cary, NC) was used to create a Best-Worst Scaling questionnaire. The respondent burden was reduced using a blocked, balanced, fractional factorial design, based on optimal D-efficiency [22]. The final design included four versions of the questionnaire, each consisting of 10 choice sets. The choice experiment was pilot tested on orthopaedic trauma patients in an outpatient setting to validate respondent comprehension and study feasibility before the final administration.

**Fig. 2:**
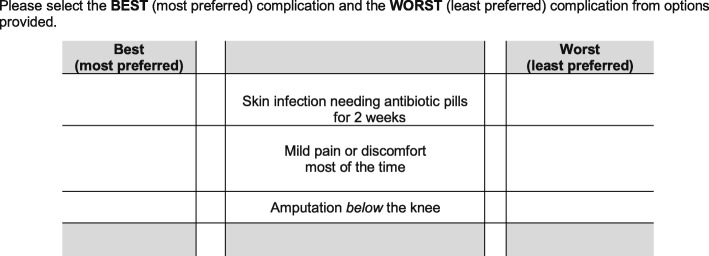
Example of a Best-Worst Scaling experiment choice set used in this study

Prior to completing the Best-Worst Scaling questionnaire, respondents answered several demographic questions and indicated which orthopaedic complications they had experienced during their post-operative clinical course. This process served to familiarize patients with the description of each attribute level prior to the choice experiment. To ensure face validity for the attribute descriptions, a chart review was performed to compare each patient’s reported post-surgical complications with any complications noted in the electronic medical records. Each choice set included a brief clinical scenario designed to establish a common context in which the post-surgical complications included in the choice sets could occur. Each choice set presented the respondent with three possible attribute levels (clinical outcomes) (see Fig. 2 for a sample choice set), and the respondents were asked to select the best and worst attribute level based on their personal preferences. This process was then repeated for the remaining choice sets (*n* = 10), with each subsequent choice set containing a different combination of the attribute levels.

### Eligibility criteria

The Best-Worst Scaling questionnaire was administered to English-speaking patients, 18 years of age or older with a surgically treated appendicular fracture from November 2017 to March 2018. Patients were enrolled in the study at an outpatient follow-up appointment, at which time they provided written informed consent and completed the written questionnaires. Electronic medical records were reviewed to assess respondent injuries, treatments, and complications. To ensure adequate statistical power for an a priori defined subgroup analysis by injury location, study participants were purposely sampled to ensure at least 50 participants with each of the following fractures: hand/wrist; upper extremity (proximal to distal ¼ radius/ulna); hip (pelvis, acetabulum, femoral neck, and greater/lesser trochanter), tibia/femur (distal to lesser trochanter and proximal to ankle fractures), and foot/ankle.

### Statistical analysis of Best-Worst Scaling data

There is no consensus on the appropriate sample size calculation for choice experiments; however, previous research recommends a minimum of 50 respondents in each sub-group included in the analysis [23]. Ten sub-groups with hypothesized divergent outcome preferences were monitored to ensure adequate representation in the sample.

The BWS statistical analyses were performed using JMP Pro Version 13 (Cary, NC). Patient demographic and clinical characteristics were described using means and standard deviations for continuous variables, and frequencies and proportions described categorical variables. A hierarchical Bayesian multinomial logit model was used to estimate the utility for each of the included clinical outcomes. This technique derives posterior estimates of the respondent’s utility based on the distribution of coefficients across the study sample and the individual respondent’s utility coefficients. Model parameters were calculated iteratively using Gibbs sampling. We ran 10,000 iterations, including 5000 burn-in iterations. The respondent-level covariates are estimated based on the algorithm described by Train, which incorporates Adaptive Bayes and Metropolis-Hastings approaches [24]. The likelihood function for the utility parameters for a given respondent is based on a model for each subject’s preference within a choice set, given the attributes in the choice set [25]. The parameters for each attribute level represent the mean of these iterations, and the utility of each included outcome estimates the strength and direction of the respondents’ preference towards a given outcome. The utility estimates for a specific outcome derived in the model have no direct interpretation, and can only be interpreted relative to another utility estimate in the model. We set the mean utility at zero for perfect health; all other possible outcomes are then presented as negative utilities.

To test heterogeneity in respondents’ utility for each included clinical outcome, ten demographic and injury-specific covariates were independently tested as interaction terms in the primary model. To adjust for ten statistical tests, we set the level of significance for the interaction terms at α =0.05/10 = 0.005. Only covariates with a significant independent interaction were jointly tested with a α = 0.005 level of significance. If a significant interaction was observed in the joint testing, a stratified analysis was performed for covariate and outcomes using a one-way analysis of variance (ANOVA) test. Significant associations between the covariates and a specific outcome at α = 0.05 in the ANOVA test were further tested using a Tukey-Kramer post hoc test (Tukey JW: The problem of multiple comparisons, Unpublished; [26]). To determine if experiencing a clinical outcome is associated with a different utility for that outcome, we stratified respondents by those who had and had not experienced the outcome. The respondent-level utilities for the outcome of interest were then compared using a Student’s t-test.

### Derivation of composite endpoint weights

An orthopaedic trauma composite endpoint weighting technique based on the mean utilities of the component outcomes and a modified version of the conditional logit formula described by McFadden [19] is provided below:

$$ {W}_a=\frac{e^{u_b}+{e}^{u_i}}{e^{u_a}+{e}^{u_b}+{e}^{u_i}} $$

The weight (*W*) is calculated separately for each included outcome *a* where *u* is the mean utility of each included outcome. *b* and *i* note the component outcomes included in the composite. A weight calculator, with sub-group adjustment, is included in the Additional file 1.

A hypothetical pilon fracture trial was used to illustrate the application of the proposed weighting technique (Table 1). In this hypothetical trial, 1000 patients are randomized to hypothetical Treatment A (*n* = 498) or Treatment B (*n* = 502). Three components (deep surgical site infection, bone healing complication, and superficial surgical site infection) were included in the hypothetical trial’s primary composite endpoint. The effect of Treatment A versus Treatment B on the composite endpoint was then calculated using several unweighted methods, including a Fisher’s Exact Test, time to first event analysis, and a random effects model. For comparison, the treatment effect was also calculated using several methods that accounted for the proposed component weights, including a Wilcoxon Rank Sums test, time to event allowing for weighted repeated events, and a random effects model that accounted for component weights [27]. The effect size for the random effects models are reported as odds ratios, and hazard ratios are used for the time to event models [28]. The Probability Index was used to report the treatment effect for the Wilcoxon Rank Sums test [27–29]. These analyses were performed using R Version 3.6.1 (Vienna, Austria). All of the data and code for the models are included in Additional files 1 and 2. However, for simplicity, only the unweighted and weighted time to event analysis are reported in the results section.

**Table 1:** Summary of events in a hypothetical pilon fracture trial

			Treatment A (*n* = 498)	Treatment B (*n* = 502)
Outcomes	Utility	Weight	Number of Events	Number of Events
Deep SSI	−5.69	0.93	61	91
Bone healing complication	−5.20	0.88	30	55
Superficial SSI	−3.29	0.20	98	42
**Total**			**168**	**174**

*SSI* Surgical site infection

## Results

### Sample characteristics

A total of 428 patients consented for the Best-Worst Scaling questionnaire at their scheduled follow up visits. Of those, 32 patients (7.5%) did not clearly indicate best and worst outcomes in the Best-Worst Scaling choice sets and were omitted from the analysis. The sociodemographic and fracture characteristics of the survey respondents are shown in Table 2. The mean age of the respondents was 48.7 years, and the respondents were more commonly male (58.3%) and white (66.4%). The median time from initial orthopaedic injury to survey completion was four months (IQR: 2–12 months). Nearly half (47.5%) of respondents had a tibia or femur fracture below the lesser trochanter. The most commonly experienced post-surgical outcome was ‘severe pain or discomfort’ (42.2%) followed by ‘bone healing complication’ (31.3%), and ‘moderate pain or discomfort’ (29.3%).

**Table 2:** Characteristics of study participants

Characteristic	*n* = 396
Age, mean (SD)	48.7 (17.5)
Sex, Male, *n* (%)	231 (58.3)
Ethnicity, *n* (%)	
White	263 (66.4)
African-American	98 (24.7)
Asian/South Asian	10 (2.5)
American Indian/Alaskan Native	10 (2.5)
Hispanic/Latino	9 (2.3)
Other	6 (1.5)
Marital Status, *n* (%)	
Single	158 (39.9)
Married	144 (36.4)
Divorced/Widowed/Separated	94 (23.7)
Education, *n* (%)	
Less than high school	50 (12.6)
High school diploma	132 (33.3)
Some college	96 (24.2)
Degree	75 (18.9)
Graduate/Professional degree	40 (10.1)
Annual Income, *n* (%)	
Less than $10,000	114 (28.8)
$10,000 - $34,999	94 (23.7)
$35,000 - $49,999	54 (13.6)
$50,000 - $74,999	57 (14.4)
$75,000 - $100,000	26 (6.6)
More than $100,000	32 (8.1)
Not reported	19 (4.8)
Heath Insurance, *n* (%)	
Medicare/Medicaid/TRICARE	207 (52.2)
Private	175 (44.2)
No insurance	14 (3.6)
Injury Location, *n* (%)^a^	
Tibia/Femur (below lesser trochanter)	188 (47.5)
Foot and ankle	107 (27.0)
Femoral neck/pelvis/acetabulum	80 (20.2)
Upper extremity (proximal to carpals)	53 (13.4)
Hand	55 (13.8)
Complications, *n* (%)^b^	
Severe pain	167 (42.2)
Bone healing complication	124 (31.3)
Moderate pain	116 (29.3)
Mild pain	98 (24.7)
Deep surgical site infection	57 (14.4)
Superficial surgical site infection	35 (8.8)
Below knee amputation	11 (2.8)
Above knee amputation	3 (0.8)

^a^Proportions exceed 100% as 73 patients had fractures is multiple anatomical locations

^b^ Proportions exceed 100% as 173 patients suffered from multiple complications

### Utilities of the clinical outcomes

The mean utility for each of the included clinical outcomes was scaled relative to “perfect health” (referenced at zero) (Table 3). Of the ten included clinical outcomes, the greatest importance was associated with death (mean utility = − 8.91, 95% CI -9.23 - -8.65), followed by an above knee amputation (AKA) (− 7.66, 95% CI -7.83 - -7.48]). Mild pain (− 3.30, 95% CI -3.46 - -3.13) and a superficial surgical site infection (− 3.29, 95% CI − 3.39 to − 3.16) were determined to be the outcomes of least importance to the respondents. The was no overlap in the confidence intervals of the clinical outcomes, except for those of superficial surgical site infection and mild pain, where considerable overlap in their utilities was observed.

**Table 3:** Utility estimates for all of the included clinical outcomes

Outcome	Mean Utility	Lower 95%	Upper 95%
Death	−8.91	− 9.23	− 8.65
Amputation [above knee]	−7.66	−7.83	−7.48
Amputation [below knee]	−6.97	− 7.14	−6.85
Severe pain	−5.90	−6.00	− 5.80
Deep surgical site infection	− 5.69	− 5.81	− 5.60
Bone healing complication	− 5.20	− 5.31	− 5.09
Moderate pain	−4.59	− 4.69	−4.57
Mild pain	−3.30	−3.46	−3.13
Superficial surgical site infection	−3.29	− 3.39	− 3.16
Perfect health	0.00	−0.37	0.44
Model Statistics			
Total iterations	10,000		
Burn-in iterations	5000		
Number of respondents	396		

### Heterogeneity in utilities of clinical outcomes

Ten covariates were independently tested as interaction terms in the primary model. There was no heterogeneity in the respondent’ mean utility of the component outcomes based on sex, time since treatment, the location of their injury, or specifically an open tibia fracture. Statistically significant interactions based on age, race, education level, income level, and health insurance status were observed. The association between these five covariates and the respondent’s utilities for the included clinical outcomes was further tested using a stratified analysis with the findings reported in Table 4.

**Table 4:** Heterogeneity in the importance of clinical outcomes by patient characteristics

Characteristic	*N*	Death	Above knee amputation	Severe pain	Deep SSI	Moderate pain	Superficial SSI
		Mean (SD)	Mean (SD)	Mean (SD)	Mean (SD)	Mean (SD)	Mean (SD)
Age							
18–64	320		−7.70 (0.17)**				
> =65	76		−7.75 (0.11)				
Race							
White	263		−7.68 (0.14)***	−5.92 (0.25)*		−4.61 (0.23)**	−3.18 (1.13)*
Non-white	133		−7.61 (0.17)	− 5.86 (0.26)		− 4.55 (0.22)	−3.48 (1.21)
Education							
High school or less	182	−8.78 (0.78)**	−7.63 (0.12)**	−5.85 (0.25)***		−4.56 (0.23)*	−3.51 (1.21)**
Some college	96	−9.00 (0.61)	−7.69 (0.14)	−5.91 (0.26)		−4.59 (0.21)	−3.10 (1.16)
Degree or higher	115	− 9.03 (0.61)	−7.68 (0.14)	−5.97 (0.26)		−4.63 (0.22)	−3.06 (1.02)
Income Level							
Less than $10,000	114			−5.86 (0.27)*	−5.70 (0.12)*	−4.57 (0.21)**	−3.41 (1.07)**
$10,000 - $34,999	94			−5.88 (0.25)	−5.70 (0.11)	−4.54 (0.21)	−3.51 (1.37)
$35,000 - $74,999	111			−5.92 (0.24)	−5.69 (0.12)	−4.61 (0.22)	−3.17 (1.08)
$75,000 or more	58			−5.98 (0.28)	−5.65 (0.09)	−4.67 (0.24)	−2.87 (1.09)
Health Insurance							
Medicare/Medicaid/TRICARE	205	−8.83 (0.76)**			−5.70 (0.11)*		
Private	175	−9.01 (0.61)			−5.68 (0.11)		

Level of significance as determined by ANOVA test. *P < 0.05, **P < 0.01, ***P < 0.001

*SSI* Surgical site infection

For each included clinical outcome, the respondent-level utilities for that specific outcome were compared between respondents that had experienced that particular outcome versus those that had not experienced the outcome. Of the 72 comparisons, only seven comparisons demonstrated significantly different mean utilities. Respondents with bone healing complications were less averse to an amputation above the knee (− 7.63 vs. -7.67, *P* = 0.02) compared to other respondents. Respondents with an above knee amputation were more averse to death (− 9.50 vs. -8.91, *P* < 0.01), but less averse to a superficial surgical site infection (− 2.07 vs. -3.29, *P* < 0.01). Respondents with a below knee amputation placed less importance on mild pain (− 3.49 vs. -3.30, *P* = 0.02) and superficial surgical site infection (− 2.66 vs. -3.30, *P* < 0.01) but a greater importance on severe pain (− 6.07 vs. 5.90, *P* = 0.04) compared to the other respondents. Respondents who experienced a superficial surgical site infection had a greater aversion to severe pain (− 5.99 vs. 5.89, *P* = 0.04).

### Composite outcome weighting: an example

**Fig. 3:**
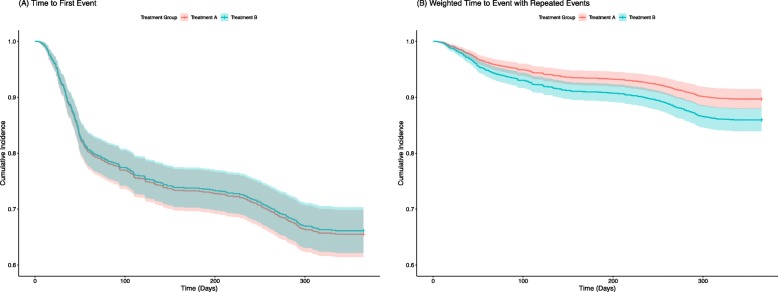
Survival curves of an unweighted time to first event analysis (**a**) and a weighted time to event analysis that allowed for repeated events (**b**) using the hypothetical pilon frature data

For the hypothetical pilon fracture trial, the results with the unweighted composite endpoint using a time to first event analysis would have determined that there was no difference between the two treatments (hazard ratio (HR): 1.02, 95% CI 0.83–1.27, *P* = 0.83) (Fig. 3). When weights are applied to the included component outcomes, and the analysis allows for patients to have more than one event, Treatment A is superior (HR: 0.72, 95% CI 0.57–0.90, *P* < 0.01). A similar difference in effect size was observed when the data were analyzed using unweighted and weighted random effects models (Additional file 3**).** However, the treatment effect was not statistically significant when the weights were applied using a global rank approach, and treatment groups were compared using a Wilcoxon Rank Sums test and Probability Index Model.

## Discussion

This study presents a novel composite endpoint weighting technique that includes ten, commonly-reported, orthopaedic trauma clinical outcomes. Hierarchical Bayesian modeling was used to calculate the importance, and heterogeneity in the importance of these outcomes in a cohort of nearly 400 orthopaedic trauma patients. Patients consistently ranked clinical outcomes according to a logical gradient ranging, from perfect health to death. Some heterogeneity in importance was observed based on respondent age, race, education level, income level, and health insurance provider. We did not observe heterogeneity in responses based on the location of the fracture or time since the initial treatment, suggesting the observed utility estimates and weighting technique has face validity across multiple fracture types and clinical experiences.

To our knowledge, this is the first study to incorporate patient preferences derived from a choice experiment into a composite endpoint weighting technique for orthopaedic outcomes. Other efforts at weighting composite endpoints have included assigning weights based on clinical and research experience [1, 8, 30], hierarchical ranking of outcomes for an entire cohort of patients in a trial [31, 32], and the inclusion of a measure of “importance to patients” assigned by clinical experts [8, 32, 33]. Outside of cardiovascular research, patient surveys on the relative value of component outcomes of composite endpoints have not been incorporated into weighing techniques [11, 12, 34, 35].

This study’s patient-centered composite endpoint weighting technique represents an improvement on previous weighted composite endpoint techniques. This work advances patient-centered outcomes research by weighting study outcomes using responses derived from the study population of interest. For the orthopaedic community, the technique provides a set of ten common clinical outcomes researchers may incorporate into future composites endpoints. The limited heterogeneity in observed preferences suggests a common value gradient for clinical outcomes that is not altered by the type of fracture, or the time since injury, and only a small variation based on outcomes experienced. Weightings may be adjusted to reflect the relative importance of an outcome of interest for specific subpopulations, when heterogeneity in that subpopulation exists on a specific outcome, such as an above knee amputation among patients over the age of 65.

Additionally, the technique addresses an important limitation of traditional composite outcomes. The weighting formula can to easily applied to several different statistical methods, including time to event analysis, multivariate modeling, or a global rank test [28, 29]. Multiple events can be included for a single patient in any of the three methods. Furthermore, multiple events per patient could be used in a time-to-event analysis enabling a comparison of the trajectory of clinical outcomes subsequent to treatment [36]. The confidence intervals associated with the mean utility of each clinical outcomes allows for a sensitivity analysis of treatment effect based on the distribution of the weightings. In the weighting formula, the weights adjust relative to the components that are included in the composite. The precision of the weights is useful in distinguishing order in a global rank test with several components of similar weight [27, 28].

Despite the strengths of this study, several limitations must be considered. This study enrolled patients from a single trauma center. While the trauma center has a statewide catchment, sample populations from other regions may vary in their relative importance for the included outcomes. Although respondents may have had a different understanding of clinical outcomes described in the survey, a comparison of patient-reported outcomes with the medical records found 96% accuracy in reporting, suggesting an adequate comprehension of the included clinical outcomes. The questionnaire’s brief descriptions of the clinical outcomes may have not adequately conveyed the magnitude of such an event for a patient and are open to subjective interpretation. However, the overall homogeneity in the importance of the clinical outcomes suggests a consistent understanding by the respondents. Finally, the list of clinical outcomes included in the study is not exhaustive. While there are many other clinical outcomes commonly reported in orthopaedic trauma research, the identification of outcomes included in this analysis was based on a synthesis of the literature and conducted in collaboration with clinical experts and orthopaedic patient trauma survivors who confirmed the proposed outcomes were both commonly used and relevant to patients. This weighting technique could be easily expanded to other outcomes and replicated in other health conditions. However, at present, the application of this weighting technique is limited to studies with component outcomes included in our model.

## Conclusion

Based on prospectively collected preference data from nearly 400 orthopaedic trauma patients, the study proposes a novel composite endpoint weighting technique. The findings suggest an overall homogeneity among orthopaedic trauma patients in their importance towards clinical outcomes. This composite endpoint technique applies weights to the component outcomes based on orthopaedic trauma patient preferences and can be applied to several types of statistical comparisons to estimate the clinical benefit of a treatment.

## Supplementary information

**Additional file 1.** Composite weighting calculator.

**Additional file 2.** Data for hypothetical pilon fracture trial available in long and wide format.

**Additional file 3.** Plausible unweighted and weighted methods of analyses for counts, time to event, and multivariate analysis.

## Data Availability

The data supporting the conclusions of this article are included as Supplementary Material within the article.

## Abbreviations

AKA: Above knee amputation
ANOVA: Analysis of variance
SSI: Surgical site infection
