# Supplementary material for: A patient-centered composite endpoint weighting technique for orthopaedic trauma research
Source: BMC Med Res Methodol. 2019 Dec 26;19:242. doi: 10.1186/s12874-019-0885-7 (PMC6933647; doi:10.1186/s12874-019-0885-7)
Supplement: Supplementary file 2 — Additional file 2. Data for hypothetical pilon fracture trial available in long and wide format. [file 12874_2019_885_MOESM2_ESM.docx]

**Supplementary Appendix A.** Composite Weighting Calculator

Please note that a composite weighting calculator has been developed using this data. However, the journal submission system does not support the .xlsm file and therefore it can be downloaded from the link below.

<https://www.dropbox.com/sh/i09nscykuhfo8fw/AAAPOKv9zG2dBEeJcspCGvRja?dl=0>
