# Supplementary material for: A patient-centered composite endpoint weighting technique for orthopaedic trauma research
Source: BMC Med Res Methodol. 2019 Dec 26;19:242. doi: 10.1186/s12874-019-0885-7 (PMC6933647; doi:10.1186/s12874-019-0885-7)
Supplement: Supplementary file 3 — Additional file 3. Plausible unweighted and weighted methods of analyses for counts, time to event, and multivariate analysis. [file 12874_2019_885_MOESM3_ESM.docx]

**Supplementary Appendix C.** Plausible unweighted and weighted methods of analyses for counts, time to event, and multivariate analysis.

**1. BIVARIATE TESTS**

**Unweighted - Fisher’s Exact Test**

A participate with any one of the three component outcomes is counted as having an event for the analysis.

**Treatment A** (n, %): 168 (34%)

**Treatment B** (n, %): 174 (35%)

**Treatment Effect:** Odds Ratio, 0.96 (95% CI: 0.73 – 1.26, p=0.79)

***R Code:***

TAB <- table(wide$Treatment, wide$Event)

fisher.test(TAB, conf.int = TRUE)

**Weighted – Wilcoxon Rank Sums Tests**

Similar to a global rank analysis, the component weights are multiplied by each outcome event and summed for each study participant. The rank sums of each Treatment group are compared using the Wilcoxon Rank Sums test with the effect determined using a Probability Index Model.

**Treatment A** (mean weight, sd): 0.21 (0.38)

**Treatment B** (mean weight, sd): 0.28 (0.44)

**Probability of Treatment Benefit** (A vs. B): 6% (95% CI: 5 – 19%), p=0.26

***R Code:***

library("pim")

wilcox.test(wide$WeightEvent ~ wide$Treatment,conf.int = TRUE)

pim1<-pim(WeightEvent ~ Treatment, data = wide)

summary(pim1)

confint(pim1)

**2. TIME TO EVENT ANALYSIS**

**Unweighted - Time to Event Analysis**

A Cox Proportional Hazard model was fit to the data. Only the first component event to occur was accounted for in the time to event analysis.

**Treatment Effect:** Hazard Ratio, 1.02 (95% CI: 0.83 – 1.27, p=0.83)


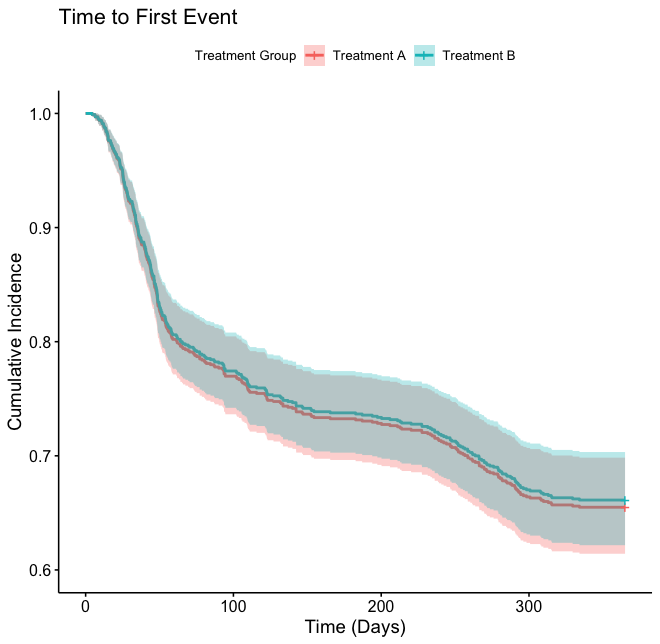


***R Code:***

library("survival")

fit3 <- coxph(Surv(EventDays, Event) ~ Treatment, data = wide)

summary(fit3)

**Unweighted - Time to Event Analysis with Repeated Events**

In this second, unweighted Cox Proportional Hazard model, multiple events were allowed in the model.

**Treatment Effect:** Hazard Ratio, 1.02 (95% CI: 0.84 – 1.24, p=0.80)

**
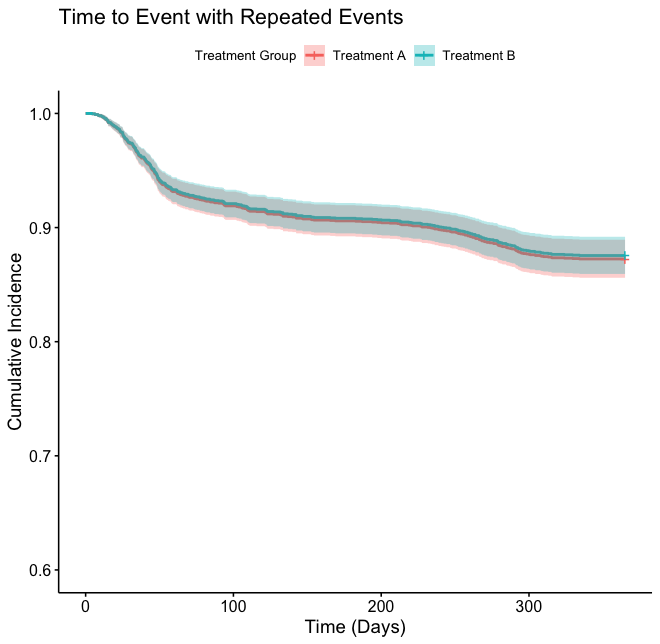
**

***R Code:***

library("survival")

fit1 <- coxph(Surv(TimetoEvent, Event) ~ Treatment + cluster (ID), data=long)

summary(fit1)

**Weighted - Time to Event Analysis with Repeated Events**

The third time to event model weighted the Cox Proportional Hazard model, and multiple events were allowed.

**Treatment Effect:** Hazard Ratio, 0.72 (95% CI: 0.57 – 0.90, p<0.01)

**
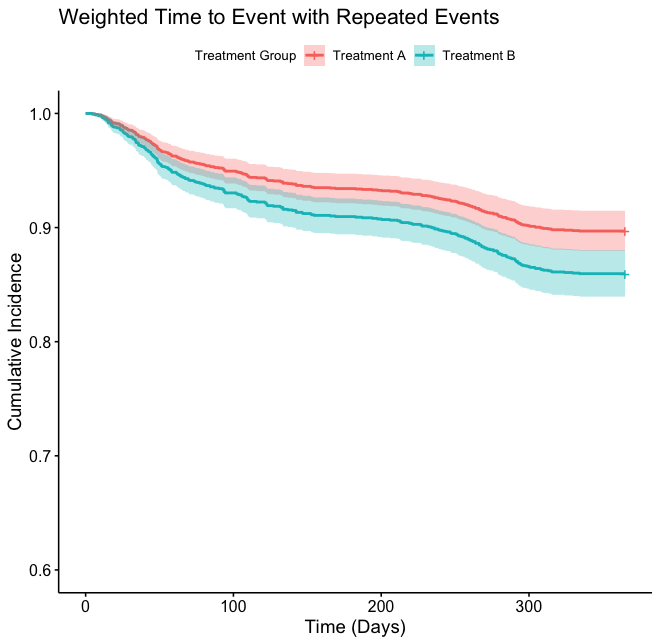
**

***R Code:***

library("survival")

fit2 <- coxph(Surv(TimetoEvent, Event) ~ Treatment + cluster (ID),weights = EventWeight, data=long)

summary(fit2)

**3. MULTIVARIATE METHODS**

**Unweighted - Random Effects Model**

The data was fit with a binomial generalized linear model that allowed for repeated events by including a random intercept for the subject ID.

**Treatment Effect:** Odds Ratio, 1.01 (95% CI: 0.81 – 1.25, p=0.93)

***R Code:***

library("lme4")

library("broom.mixed")

fit4<-glmer(Event ~ Treatment + (1|ID), family = binomial, data=long, nAGQ = 9)

summary(fit4)

tidy(fit4,conf.int=TRUE, exponentiate = TRUE, effects="fixed")

**Weighted - Random Effects Model**

The data was fit with a binomial generalized linear model that allowed for repeated events by including a random intercept for the subject ID.

**Treatment Effect:** Odds Ratio, 0.70 (95% CI: 0.53 – 0.91, p<0.01)

***R Code:***

library("lme4")

library("broom.mixed")

fit5<-glmer(Event ~ Treatment + (1|ID), family = binomial, weights = EventWeight, data=long, nAGQ = 9)

summary(fit5)

tidy(fit5,conf.int=TRUE, exponentiate = TRUE, effects="fixed")
